# Epidemiological Indicators of SARS-CoV-2 (COVID-19) and Vaccination Effectiveness on the Report of Positive Cases in the Colombian Army

**DOI:** 10.3389/fmed.2021.791761

**Published:** 2021-12-09

**Authors:** Maria Clara Duque, Camilo A. Correa-Cárdenas, Sebastián Londoño-Méndez, Carolina Oliveros, Julie Pérez, Carlos D. Daza, Lorena Albarracin, Elizabeth K. Márquez, Maria T. Alvarado, Frank De Los Santos Ortíz, Yanira Romero, Sergio Gutierrez-Riveros, Claudia Méndez

**Affiliations:** ^1^Laboratorio de Referencia e Investigación, Grupo de Investigación en Enfermedades Tropicales del Ejército (GINETEJ), Dirección de Sanidad Ejército, Bogotá, Colombia; ^2^Semillero Observatorio para la contención del COVID-19 en América Latina, Universidad del Rosario, Bogotá, Colombia; ^3^Trainee Field Epidemiology Training Program (FETP), Training Programs in Epidemiology and Public Health Interventions Network (TEPHINET), Bogotá, Colombia

**Keywords:** SARS-CoV-2, molecular and antigen diagnostic tests, epidemiology, military forces, Colombia, mortality rate, case fatality rate, attack rate

## Abstract

The description of the epidemiological indicators of SARS-CoV-2 (COVID-19), such as the mortality rate (MR), the case fatality rate (CFR), and the attack rate (AR), as well as the geographical distribution and daily case reports, are used to evaluate the impact that this virus has had within the Colombian Army and its health system. As military forces around the world represent the force that defends sovereignty, independence, the integrity of the national territory, and the constitutional order, while maintaining migration controls in blocked border areas during this critical pandemic times, they must carry out strict epidemiological surveillance to control the situation among the servicemen. Up to date, the Colombian Army has faced a very high attack rate (AR = 8.55%) due, among others, to living conditions where active military personnel share bedrooms, bathrooms, and dining facilities, which facilitate the spread of the virus. However, being a mainly young and healthy population, the MR was 1.82 deaths/1,000 ha, while the CFR = 2.13% indexes consistently low if compared with those values reported for the national population. In addition, the effectiveness of vaccination is shown in daily cases of COVID-19, where, for the third peak, the active military population presented a decrease of positive patients compared to the dynamics of national transmission and the total population of the military forces (active, retired, and beneficiaries).

## Introduction

COVID-19, the disease caused by the SARS-CoV-2 virus that first emerged in the city of Wuhan, Hubei Province—China, was declared a public health emergency of international concern on January 30, 2020, after 18 countries besides China reported a total of 98 cases of the novel coronavirus. On March 11, 2020, the World Health Organization proclaimed the COVID-19 a pandemic due to the rapid levels of propagation and the severity of the disease caused by the SARS-CoV-2 virus (1). In Colombia, the first COVID-19 confirmed case was reported on March 6, 2020, in a Colombian citizen returning from Italy (2). Since then, and due to the rapid and evolving situation of transmission, the Colombian government declared a whole-wide-country lockdown on March 22 that lasted until May 25 (3) to later become flexible and selective.

Inside the military forces, the Colombian Army through its health directorate issued guidelines and epidemiological alerts (4), according to other governments (5), foreign military strategies (6, 7) as well as the country's epidemiological situation (8, 9), to all the military and tactic units. Alongside, the Reference and Research Laboratory of the Army's Health Directorate was conditioned and nationally certified to respond to the diagnostic needs of the rapidly evolving pandemic on April 27. Since then, this laboratory has processed nationwide patient samples from the army, navy, and air force troops together with their immediate families, the military hospital patients, and retired military, being of paramount relevance for the country's military forces.

According to the operational health office from the Army's Health Directorate, the first reported case of COVID-19 was a retired military at Ibagué, Tolima, on March 15, 2020, followed by an active-duty soldier infected in Bogotá with a positive diagnostic test on March 23, 2020. Since then, and until June 30, 2021, dates defined in this study, we have constantly diagnosed and carried out an epidemiological follow-up on patients, and, herein, we aim to estimate the epidemiological indicators of SARS-CoV-2, determine the geographic distribution and demographic outlook of confirmed cases, and report the daily SARS-CoV-2 cases by test date in the Colombian Army Health System.

## Methods

### Ethics Statement

This study was done following the Declaration of Helsinki and its later amendments. The Research Ethics Committee of the Universidad del Rosario, Bogotá, approved the research study subjected to this publication under Act DVO005-1508-CV1400 of April 8, 2021. This committee is governed by the legal and ethical guidelines of Colombia through resolutions 8,430 from 1993 and 2,378 from 2008 of the Health and Social Protection Ministry.

The data of the patients were anonymized to obtain all epidemiological indicators (Dataset S1). The patients provided oral and written informed consent.

### Molecular and Serological Tests

To obtain a positive diagnosis for SARS-CoV-2 by Real-Time Reverse Transcription Polymerase Chain Reaction (RT-qPCR), oropharyngeal and nasopharyngeal sampling was done by placing a swab into a viral transportation medium LABG&M (Microgen Ltd., Colombia). Ribonucleic acid (RNA) was extracted automatically using the ab-Aid Virus RNA Extraction kit (Xiamen Zeesan Biotech Co., Ltd, China) and Lab-Aid 824s Nucleic Acid Extraction System (Xiamen Zeesan Biotech Co., Ltd, China), following the manufacturer's recommendations to obtain 100 μL as final elution of RNA.

Five molecular markers (target genes included *E, RdRP, S*, and *N*, in addition to the *RP-IC* as an exogenous internal control) were amplified in a multiplex RT-qPCR using the Allplex™ SARS-CoV-2 assay (Seegene Inc, Republic of Korea), following the manufacturer's instructions with a CFX96™ Real-Time PCR detection system (Bio-Rad Laboratories, USA). Real-time data analysis was performed using the Seegene SARS-CoV-2 Viewer Software version 3.

Besides, rapid antigen tests STANDARD^TM^ Q COVID-19 Ag Test (SD Biosensor Inc., Republic of Korea) allowed the diagnosis of positive patients as per the kit insert.

### Mapping and Graphics of Dataset

All graphical representations were performed in Tableau software v. 2021.3 (10), while mapping of the geographic distribution of COVID-19 cases was done in Mapbox © OpenStreetMap contributors, licensed under the Open Data Commons Open Database License (ODbL) by the Open Street Map Foundation (OSMF) using a Colombian departments layer.

The entire dataset of patients was obtained from a cross between the SARS-CoV-2 molecular diagnosis database of the Army's Health Directorate and the external network database SisMuestras COVID- 19 (https://apps.ins.gov.co/sismuestras) managed by the National Institute of Health of the Ministry of Health and Social Protection of Colombia, which contains all the patients reported as positive in other healthcare establishments outside the Colombian Army health department.

## Results

Since the Army's Health Directorate laboratory was endorsed to carry out the diagnosis, a total of 111,735 RT-qPCR tests have been performed in 15 months of surveillance for all the military health subsystems, which comprise all servicemen, their immediate families (beneficiaries), and retired military. Of them, a total of 41,953 samples were reported positive.

Up to June 30, 2021, a total of 44,219 cases had been diagnosed inside the Army, concerning all active, their beneficiaries, and retired populations (Table 1). Of those, 23,817 cases in servicemen corresponded to the 14.46% of the active Army troops.

**Table 1:** Number of SARS-CoV-2 confirmed infections in Colombia's Army Health System.

**Diagnostic test**	**Place**	**Number of positive results**
RT-qPCR	Army's Health Directorate Reference and Research Laboratory	32,349
	External Laboratories	6,948
Antigen (immunochromatography RDT)	Army's Health Directorate Reference and Research Laboratory and Operational Health Units in all departments of Colombia	4,922

**Figure 1:**
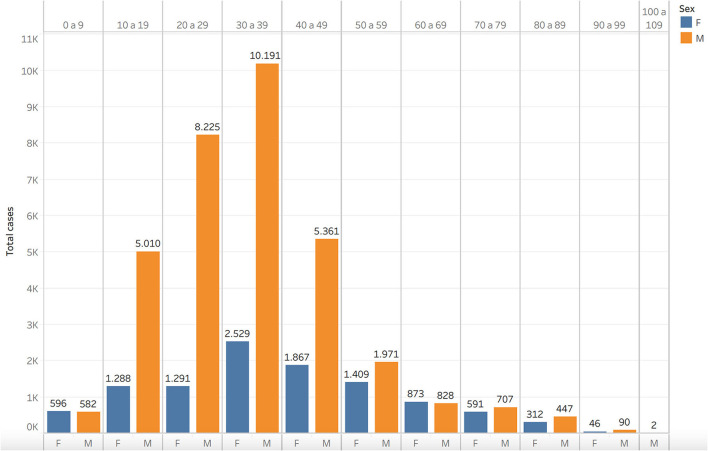
SARS-CoV-2 cases by age and sex in Colombian Army.

To this date, of the total army cases, 43,028 of the patients have recovered, and, sadly, 943 have died from COVID-19-related complications, of which 55.78% occurred in retirees, 37.54% in the beneficiaries' group, and only 6.68% in the active personnel. Thus, establishing a mortality rate MR = [number of deaths/total army health system population] ^*^ 1,000 ha = 1.82 deaths per thousand individuals and a case fatality rate or CFR = [number of deaths/number of confirmed positive cases] ^*^ 100 = 2.13%, whereas Colombia's CFR is 2.5%. However, when demographics are considered the mean age of cases among the army health system is 34-year-old, being the age groups of 20–29 and 30–39 years, the most affected ones. Accordingly, the male population is the most affected one with 75.6% of the cases (Figure 1).

Up to this point, a proper attack rate of COVID-19 inside the army could not be calculated due to the lack of reports from mild and asymptomatic cases without positive tests results and the absence of a complete serological dataset. Nonetheless, an attack rate, AR = [number of confirmed positive cases/total army health system population] ^*^ 100 = 8.55% was obtained regarding only the confirmed cases among the army.

**Figure 2:**
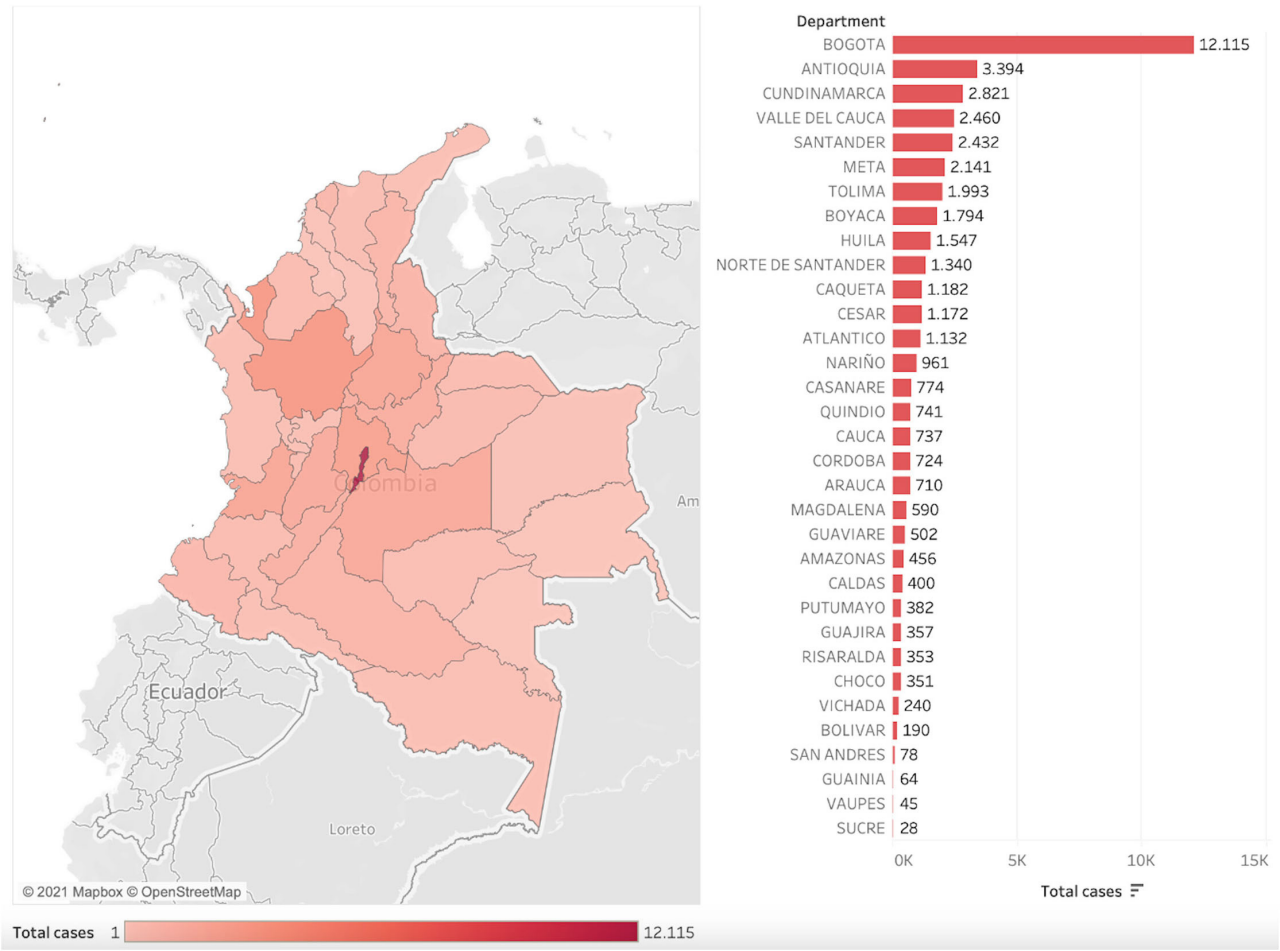
Geographic distribution and the total number of confirmed SARS-CoV-2 cases among the Colombian Army Health System by the department.

From the total army health system-infected population, 27.4% of the cases were reported in Colombia's capital district, Bogota. Followed by departments: Antioquia (7.7%), Cundinamarca (6.4%), Valle del Cauca (5.6%), and Santander (5.5%). The 47.4% of cases left were confirmed among the remaining 28 departments (Figure 2). Only 13 cases were reported among military personnel on active duty outside the country.

**Figure 3:**
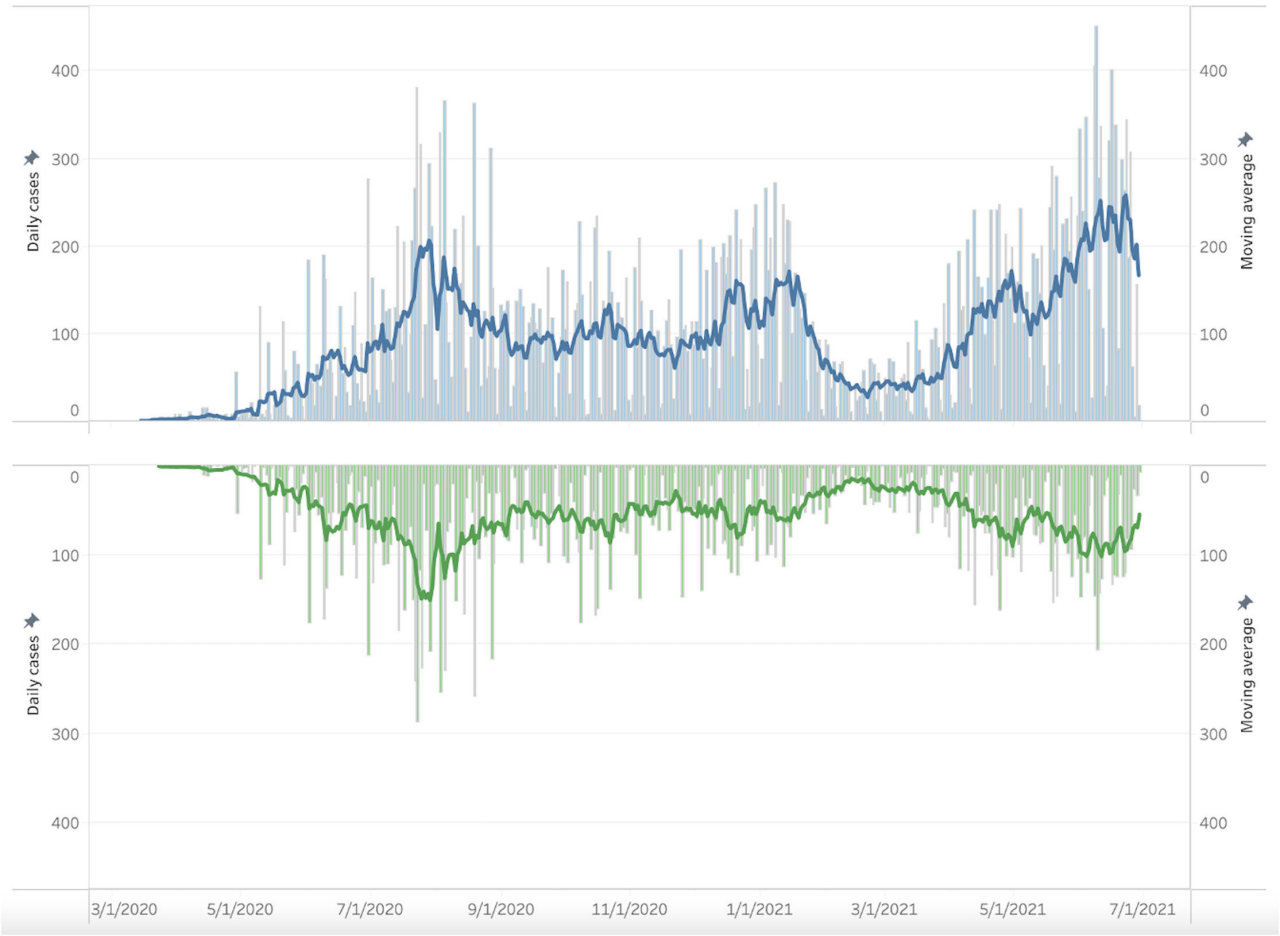
Daily SARS-CoV-2 cases by test date in the Colombian Army Health System, from March 2020 to June 2021. In blue is the whole Army health system population; in green, active-duty personnel.

Figure 3 (blue) shows the behavior of the active COVID-19 cases from March 2020 through June 2021 among the army health system (servicemen, their immediate families, and retired military included). It properly depicts the situation presented in the country. From February to May 2021, the lowest number of cases was seen inside the active military personnel, mirroring the national behavior of the disease in Colombia, but unfortunately giving way to the third most important and deadly peak in the country where the military population, as well as the army's health system beneficiaries, suffered the burden of the disease. However, it can be observed that the third peak into the epidemiological curve was less intense, and the valleys were more prolonged among active-duty personnel (Figure 3, green).

## Discussion

The Colombian army force seems to be the most affected one, among the three military forces. However, it must be considered that the army represents 80% of the total military forces' population, with 164,692 militaries, followed by the navy, with 30,426 (15%), and the air force, with 9,591 (5%) men, explaining the higher number of cases. As expected, the army's retired personnel, as well as the beneficiaries, played an important role in the number of cases presented. Yet, the epidemiological behavior of their infections is a clear representation of the country's situation as has been reported by the Ministry of Health and Social Protection through the National Institute of Health (11).

All the data hereby presented can be explained by the constitutional mission and protective duties of the military institution. Among Colombian government's first response to the pandemic, international border crossings with neighboring countries, such as Brazil, Ecuador, and Venezuela, were closed and secured by deploying military personnel, who become greatly exposed to the virus as occurred with outbreaks in Ipiales (Nariño), Leticia (Amazonas), and Arauca (Arauca) into the Colombian Army according to data of the Reference and Research Laboratory into the Army's Health Directorate. Furthermore, antinarcotic and counterinsurgency actions continued to be carried out during the health crisis, exposing those present in the scenes of the military operations.

Although an exact attack rate of COVID-19 in the army health system could not be obtained, it, clearly, must be higher than the 8.55% reported for the confirmed cases (12). One must take into that active military personnel lives quartered in military forts or deploys in groups across the country, allowing rapid transmission of the virus in comparison with an incidence and prevalence of COVID-19 in studies with civil people (13). Opposite, the age range of the troops sets the personnel in a low-risk group to develop severe forms of the disease, explaining the lower fatality rate if compared to the one presented by the civil Colombian population. This was consistent with other military epidemiological surveillance studies conducted in countries of our region, such as Bolivia, Brazil, and the USA (13–15).

Among the positive infection events that were reported in military personnel outside the country, one must highlight the case of three officials who got infected while on service at the Sinai Peninsula before their immediate return to the country after a year and a half of deployment. They completed the quarantine in Egypt, but the other 126 militaries from the platoon with negative results returned to the country on April 6, 2021. By April 22, all the returnees tested positive for SARS-CoV-2. Due to the rapid onset of the infection in this military personnel, a study was carried out, and it was determined that the SARS-CoV-2 lineage B.1.1.7, also known as the variant of concern Alpha, was responsible for these infections (16).

At last, the observed epidemiological curve differences between the active military personnel (Figure 3, green) and the total army health system population (Figure 3, blue) are most likely due to the priority vaccination program authorized by the Ministry of Health and Social Protection of Colombia. By June 2021, the start month of the greatest peak in Colombia, more than 20.3% of the active military population had completed their vaccination scheme, and 57% had, at least, one dose, according to reports of the Operational Health office of the Army's Health Directorate, serving as evidence of vaccination effectiveness and motivation for those who have not yet received the vaccine.

Concerning the herd immunity threshold, records of the Operational Health office of the Army's Health Directorate showed 65.8% of the active military personnel had a complete vaccination scheme, up to October 22, 2021, meaning that on-duty personnel of the army are above 80.26% of collective immunity if considered the 14.46% of confirmed positive patients for COVID-19. In contrast, at the same date, only 33.11% of the beneficiary and retired personnel of the Colombian Army had completed the vaccination plan, so it is estimated that they have not reached yet the herd immunity threshold.

This study presents some limitations, depicting the real situation of COVID-19 inside Colombia's military forces, considering the clinical manifestations of the disease. Even though some of the results from the molecular tests hereby reported were performed during sentinel surveillance studies, most of the asymptomatic carriers lacking a diagnosis did not enter the dataset of COVID-19 army patients, while the patients with reinfections and/or viral persistence that corresponded to <0.1% (17) were included as new individuals within the dataset, leading to some deviations in our epidemiological indicators.

In conclusion, COVID-19 management has been challenging around the world, and the situation inside the Colombian Military Forces has not been the exception. Outbreaks management inside military forts proved to be demanding due to the great number of people gathered in limited accommodations and the troops' way of life. However, the actions and guidelines provided by the Army's Health Directorate demonstrated to be effective with only a few outbreaks of concern across the country. On-time detection of cases and rapid reaction approaches were possible—thanks to the labor of the Army's Reference and Research Laboratory, which provided opportune and accurate results, without relaying the diagnosis in external laboratories already at their maximum capacity. These timely results served as a solid base for decision-making committees and epidemiological surveillance in the force.

## Data Availability Statement

The original contributions presented in the study are included in the article/Supplementary Material, further inquiries can be directed to the corresponding author.

## Ethics Statement

The studies involving human participants were reviewed and approved by the Research Ethics Committee of the Universidad del Rosario, Bogotá Colombia. Written informed consent to participate in this study was provided by the participants' legal guardian/next of kin.

## Author Contributions

MD and CM conceived and designed the study. MD, CC-C, CO, MA, FO, YR, and CM managed the funding acquisition and formulated the needs plan for the research project subjected to the current study. MD, CC-C, CO, JP, CD, LA, EM, MA, FO, YR, SG-R, and CM conducted molecular tests, reported data, and did epidemiological monitoring. MD, CC-C, SL-M, LA, and EM analyzed the data. MD and CC-C wrote the original draft of the manuscript. CC-C, SL-M, CO, JP, CD, LA, EM, MA, FO, YR, SG-R, and CM reviewed and edited the written manuscript. All authors have read and approved the final version of the manuscript.

## Funding

This article was funded by the General Directorate of Military Health (DIGSA) and Army Health Directorate (DISAN) as part of the Ministry of Defense, Colombia, through contracts-commission Nos. 181 and 209 for the execution of the research projects derived from the diagnosis of SARS-CoV-2 (COVID-19), including reagents for RT-qPCR, rapid antigen tests, research equipment, transportation of samples nationwide, and remodeling of the laboratory infrastructure.

## Conflict of Interest

The authors declare that the research was conducted in the absence of any commercial or financial relationships that could be construed as a potential conflict of interest.

## Publisher's Note

All claims expressed in this article are solely those of the authors and do not necessarily represent those of their affiliated organizations, or those of the publisher, the editors and the reviewers. Any product that may be evaluated in this article, or claim that may be made by its manufacturer, is not guaranteed or endorsed by the publisher.
